# Overexpression of PARP is an independent prognostic marker for poor survival in Middle Eastern breast cancer and its inhibition can be enhanced with embelin co-treatment

**DOI:** 10.18632/oncotarget.26470

**Published:** 2018-12-18

**Authors:** Abdul Khalid Siraj, Poyil Pratheeshkumar, Sandeep Kumar Parvathareddy, Sasidharan Padmaja Divya, Fouad Al-Dayel, Asma Tulbah, Dahish Ajarim, Khawla S. Al-Kuraya

**Affiliations:** ^1^ Human Cancer Genomic Research, King Faisal Specialist Hospital and Research Center, Riyadh, Saudi Arabia; ^2^ Department of Pathology, King Faisal Specialist Hospital and Research Centre, Riyadh, Saudi Arabia; ^3^ Department of Oncology Centre, King Faisal Specialist Hospital and Research Centre, Riyadh, Saudi Arabia

**Keywords:** PARP, XIAP, breast cancer, olaparib and embelin

## Abstract

Patients with aggressive breast cancer (BC) subtypes usually don’t have favorable prognosis despite the improvement in treatment modalities. These cancers still remain a major cause of morbidity and mortality in females. This has fostered a major effort to discover actionable molecular targets to treat these patients. Poly ADP ribose polymerase (PARP) is one of these molecular targets that are under comprehensive investigation for treatment of such tumors. However, its role in the pathogenesis of BC from Middle Eastern ethnicity has not yet been explored. Therefore, we examined the expression of PARP protein in a large cohort of over 1000 Middle Eastern BC cases by immunohistochemistry. Correlation with clinico-pathological parameters were performed. Nuclear PARP overexpression was observed in 44.7% of all BC cases and was significantly associated with aggressive clinico-pathological markers. Interestingly, nuclear PARP overexpression was an independent predictor of poor prognosis. PARP overexpression was also directly associated with XIAP overexpression, with PARP and XIAP co-expression in 15.8% (159/1008) of our cases. We showed that combined inhibition of PARP by olaparib and XIAP by embelin significantly and synergistically inhibited cell growth and induced apoptosis in BC cell lines. Finally, co-treatment of olaparib and embelin regressed BC xenograft tumor growth in nude mice. Our results revealed the role of PARP in Middle Eastern BC pathogenesis and prognosis. Furthermore, our data support the potential clinical development of combined inhibition of PARP and XIAP, which eventually could extend the utility of olaparib beyond BRCA deficient cancer.

## INTRODUCTION

Breast cancer is a leading cause of morbidity and mortality in women worldwide. In Saudi Arabia, BC is the most common cancer among females, accounting for 28.7% of newly diagnosed female cancers and the incidence continues to increase every year [1]. BC is a heterogeneous disease containing several subgroups with molecular signature [2]. Triple negative breast cancer (TNBC) is the most aggressive histological subtype of BC representing 15–20% of all BCs with a high potential for metastasis and resistance to standard therapies [3, 4]. Therefore, identifying molecular therapeutic target for aggressive and metastatic BC is warranted.

Poly (ADP-ribose) polymerases (PARPs) are a family of enzymes that share a catalytic PARP homology domain and the ability to poly (ADP-ribosyl)ate protein substrate [5, 6]. PARP proteins involved in a number of cellular processes including transcriptional regulation, DNA repair, cell survival, cell division, apoptosis, maintenance of genomic stability and telomere integrity [7, 8]. PARP-1 is the most abundant member as well as best-characterized DNA repair enzyme of PARP super family and is responsible for the majority of PARP activity in the cell [9]. PARP-1 protein overexpression has been reported in various human malignancies, including BC [10–14]. PARP inhibitors target DNA repair defects in hereditary BC [15]. PARP inhibitors as monotherapy or in combination therapy have yielded promising results against different cancers in recent clinical trials [16]. Olaparib is an orally active PARP inhibitor that selectively kills cancer cells with deficient *BRCA1* and *BRCA 2*, which encode proteins known to function in DNA repair through homologous recombination (HR) [17, 18]. However, *BRCA* – mutant tumors represent only a small fraction (2–3%) of all BCs [19] and only 12.5% of TNBCs [20], which might limit the therapeutic use of PARP inhibitor monotherapies.

X-linked inhibitor of apoptosis protein (XIAP) has been found to be a promising therapeutic molecular target in Middle Eastern BC and other cancers [21–23]. The main role of XIAP is to disrupt and inhibit apoptosis by acting at caspase 3 and -7 via the second BIR domain and caspase 9 via the third BIR domain [24–26]. We have demonstrated previously the efficacy of non-peptide small molecule inhibitor embelin on inducing apoptosis in BC cell line by binding to the BIR3 domain of XIAP and blocking the interaction of XIAP with caspase to promote apoptosis [21]. Although many combination therapies involving PARP inhibitors are being investigated [27–30], the effect of co-targeting both PARP and XIAP in BC has not yet been explored.

In this study, we first investigated the expression of PARP protein in more than 1000 Middle Eastern BC cases and their clinico-pathological correlations including patient survival. Then, we were able to demonstrate the superiority and synergism of inhibition of PARP (using olaparib) and XIAP (using embelin) together over using single inhibitor alone. This synergistic effect on cell proliferation, apoptosis and tumor growth is demonstrated both *in vitro* and *in vivo*. These data clearly demonstrate that PARP plays a significant role in the Middle Eastern BC pathogenesis and its combined inhibition with XIAP may expand the role of PARP inhibition therapy beyond *BRCA*-deficient BCs.

## RESULTS

### PARP over-expression in BC clinical cases and association with clinico-pathological parameters

**Figure 1:**
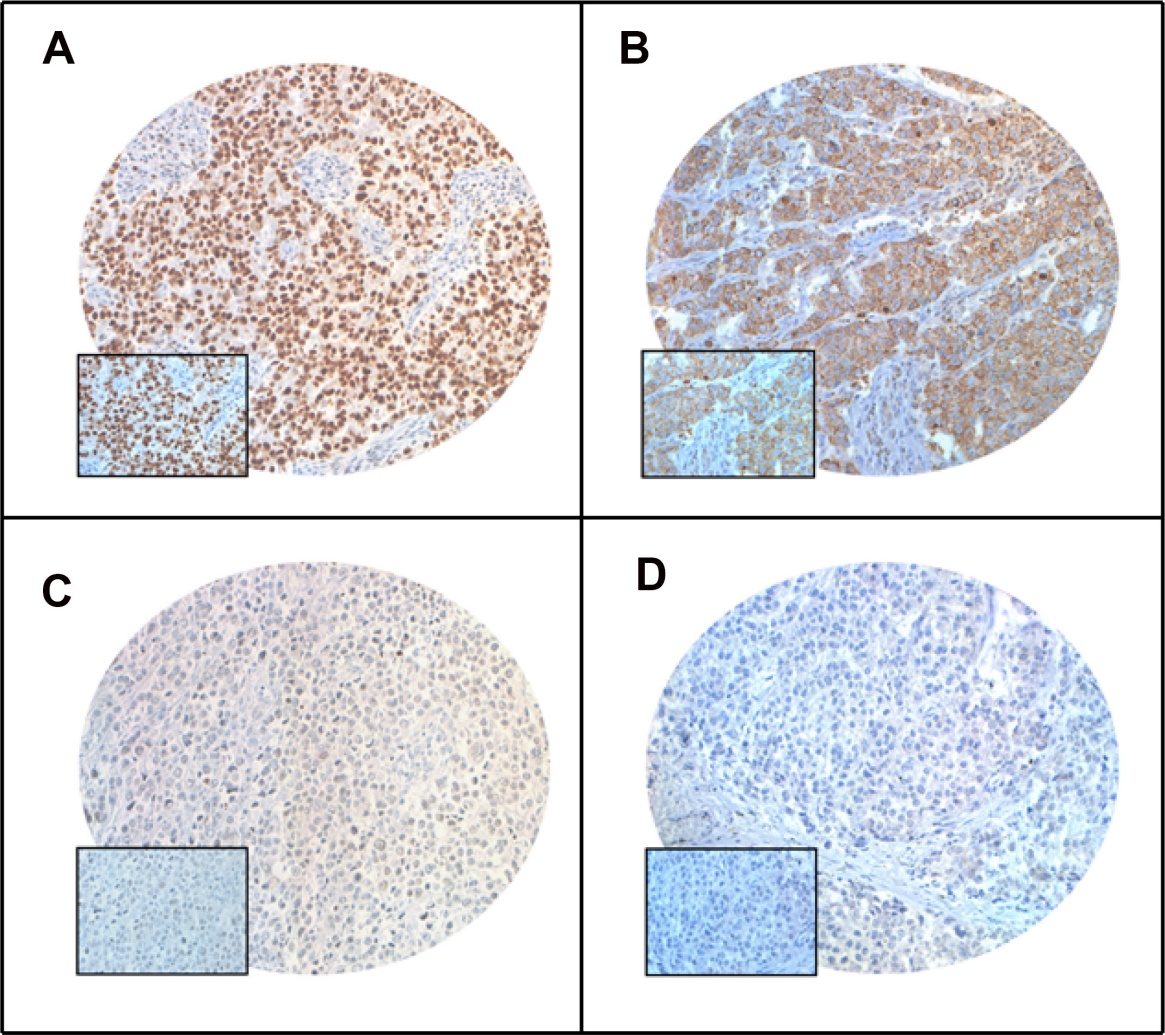
Tissue microarray based immunohistochemistry analysis of PARP and XIAP in breast cancer (BC) patients BC TMA spots showing overexpression of PARP (**A**) and XIAP (**B**). In contrast, another set of TMA spots showing reduced expression of PARP (**C**) and XIAP (**D**). 20 X/0.70 objective on an Olympus BX 51 microscope. (Olympus America Inc, Center Valley, PA, USA) with the inset showing a 40× 0.85 aperture magnified view of the same TMA spot.

PARP expression was analysed in 1008 breast cancer cases using tissue microarray. Nuclear PARP over-expression was noted in 44.7% (451/1008) of the cases (Table 1, Figure 1). PARP over-expression was associated with aggressive clinical parameters such as larger tumor size (*p* = 0.0136), distant metastasis (*p* = 0.0003), stage IV tumors (*p* = 0.0006), grade 3 tumors (*p* < 0.0001) and triple negative breast cancers (*p* < 0.0001). PARP over-expression was also found to be associated with proliferative marker Ki-67 (*p* < 0.0001) and anti-apoptotic marker XIAP (*p* < 0.0001). More importantly, PARP over-expression was significantly associated with poor 5-year overall survival (*p* = 0.0006) (Table 2, Supplementary Figure 1). On multivariate analysis, using Cox proportional hazards regression model, we found that PARP over-expression was an independent prognostic factor (HR = 1.43; 95% CI = 1.01–2.04; *p* = 0.0428) (Table 2). Interestingly, PARP and XIAP were co-expressed in 15.8% (159/1008) of our cases.

**Table 1:** Correlation of PARP protein expression with clinico-pathological parameters in breast cancer

	Total		High expression		Low expression		*p* value
	*N*	%	*N*	%	*N*	%	
Total number of cases	1008		451	44.7	557	55.3	
**Age Groups**							
≤ 50	686	68.1	305	44.5	381	55.5	0.7931
>50	322	31.9	146	45.3	176	54.7	
**Tumor size**							
T1	219	22.2	82	37.4	137	62.6	0.0136
T2	495	50.3	218	44.0	277	56.0	
T3	145	14.7	75	51.7	70	48.3	
T4	126	12.8	66	52.4	60	47.6	
**Lymph Nodes**							
N0	312	33.0	133	42.6	179	57.4	0.6011
N1	305	32.3	134	43.9	171	56.1	
N2	197	20.8	87	44.2	110	55.8	
N3	131	13.9	65	49.6	66	50.4	
**Metastasis**							
M0	917	91.0	394	43.0	523	57.0	0.0003
M1	91	9.0	57	62.6	34	37.4	
**Tumour Stage**							
I	87	9.0	32	36.8	55	63.2	0.0006
II	407	42.1	165	40.5	242	59.5	
III	381	39.5	177	46.5	204	53.5	
IV	91	9.4	57	62.6	34	37.4	
**Histological Grade**							
Well differentiated	81	8.1	21	25.9	60	74.1	<0.0001
Moderately differentiated	511	51.3	205	40.1	306	59.9	
Poorly differentiated	405	40.6	224	55.3	181	44.7	
**Histology**							
Infiltrating Ductal Carcinoma	917	93.7	421	45.9	496	54.1	0.2681
Infiltrating Lobular	46	4.7	16	34.8	30	65.2	
Mucinous Ca	16	1.6	6	37.5	10	62.5	
**Lymphovascular invasion**							
Yes	373	41.7	173	46.4	200	53.6	0.6135
No	521	58.3	233	44.7	288	55.3	
**Triple Negative**							
No	851	85.1	348	40.9	503	59.1	< 0.0001
Yes	149	14.9	101	67.8	48	32.2	
**Ki-67 IHC**							
High	624	63.5	331	53.0	293	47.0	<0.0001
Low	358	36.5	112	31.3	246	68.7	
**XIAP**							
High	284	29.5	159	56.0	125	44.0	<0.0001
Low	680	70.5	276	40.6	404	59.4	
Survival							
OS 5 Years				73.1		85.6	0.0006

**Table 2:** Univariate and multivariate analysis of PARP using Cox proportional hazard model

Clinical parameters	Univariate		Multivariate	
	Risk ratio (95% CI)	*p* value	Risk ratio (95% CI)	*p* value
**Age** Above > 50	0.93 (0.67–1.28)	0.6678	0.84 (0.58–1.18)	0.3158
**Stage** IV	7.04 (4.96–9.85)	<0.0001	4.85 (3.31–7.10)	<0.0001
**Grade** Poorly Diff.	1.56 (1.16–2.10)	<0.0001	1.38 (0.97–1.94)	0.0667
LVI	0.46 (0.33–0.63)	<0.0001	0.47 (0.34–0.66)	<0.0001
TNBC	1.82 (1.24–2.59)	0.0027	1.39 (0.89–2.11)	0.1379
PARP (High expression)	1.69 (1.26–2.29)	0.0005	1.43 (1.01–2.04)	0.0428

### Olaparib and embelin synergistically induced apoptosis in BC cells

**Figure 2:**
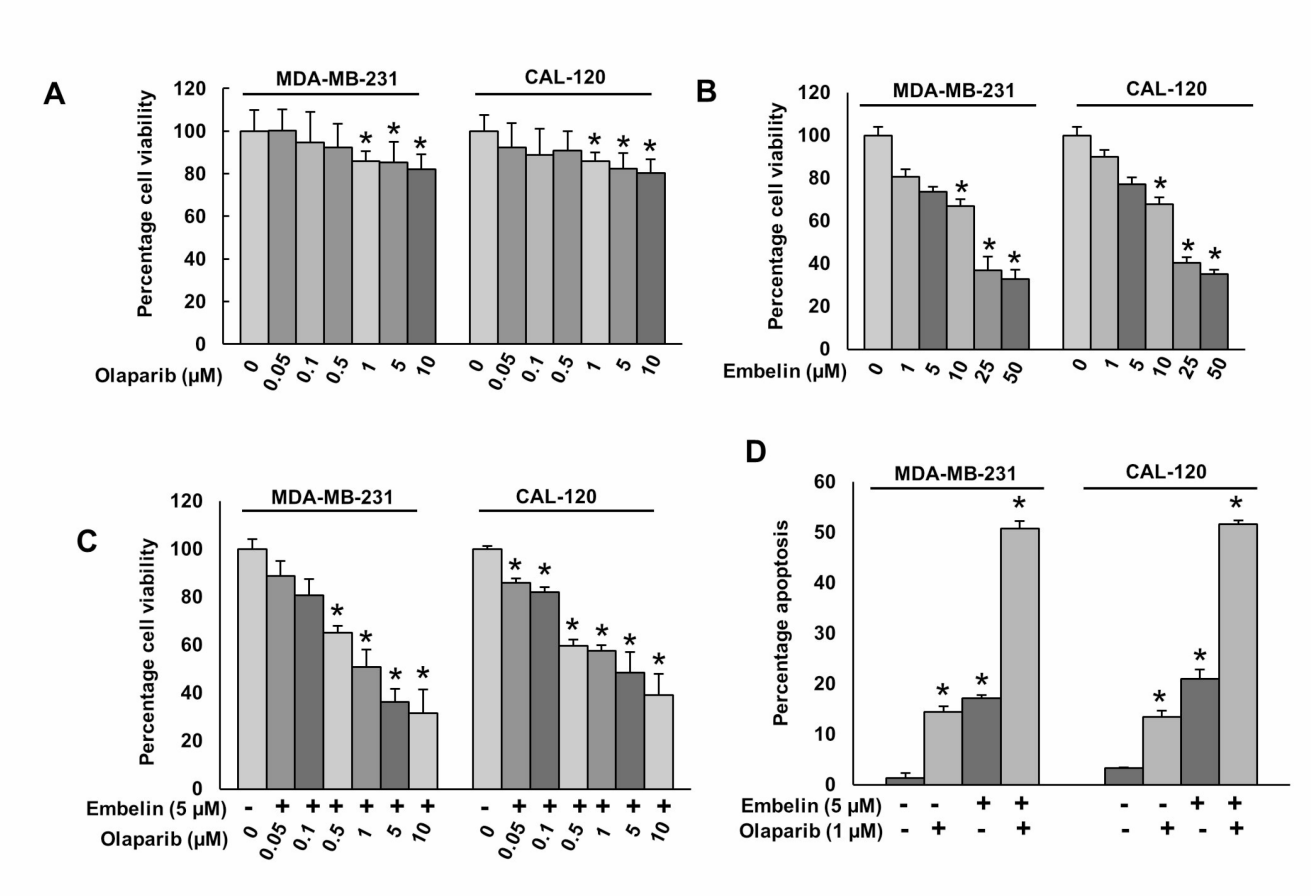
Olaparib and embelin induces synergistic apoptotic response in BC cells (**A**) Effect of olaparib on BC cell viability. BC cells were incubated with indicated doses of olaparib for 48 hours. Cell viability was performed using MTT. (**B**) Effect of embelin on BC cell viability. BC cells were incubated with indicated doses of embelin for 48 hours. Cell viability was performed using MTT. (**C**) Synergistic effect of olaparib and embelin on BC cell viability. BC cells were incubated with indicated doses of olaparib and embelin either alone or combination for 48 hours. Cell viability was performed using MTT. (**D**) Synergistic effect of olaparib and embelin on inducing apoptosis in BC cells. BC cells were incubated with indicated doses of olaparib and embelin either alone or combination for 48 hours and cells were stained with flourescein-conjugated annexin-V and propidium iodide (PI) and analyzed by flow cytometry. Data presented in the bar graphs are the mean±SD of three independent experiments. ^*^Indicates a statistically significant difference compared with control with *p* < 0.05.

Our clinical data showed that PARP overexpression was directly associated with the overexpression of anti-apoptotic protein, XIAP. Therefore, we sought to determine whether co-targeting of PARP and XIAP using specific pharmacological inhibitors, could inhibit BC growth *in vitro*. BC cells were incubated with and without indicated doses of olaparib or embelin for 48 hours; cell viability was determined by MTT assay. Olaparib treatment caused mild effect on cell viability (Figure 2A), whereas embelin alone showed a dose-dependent inhibition of cell viability in BC cells (Figure 2B). Combination of different doses of olaparib with sub-optimal dose of embelin synergistically inhibited cell viability in BC cells (Figure 2C). Using Chou and Talalay method and Calcusyn software [31], we found that olaparib at 1 μM and embelin at 5 μM had a combination Index (CI) of 0.298 (strong synergism) in MDA-MB-231 cell line (Supplementary Figure 2) and 0.358 (synergism) in CAL-120 cell line (Supplementary Figure 3) suggesting a synergistic inhibition of cell viability. We selected these doses for further experimentations. Next, to determine whether inhibition of cell viability was due to induction of apoptosis, BC cells were treated with olaparib and embelin either alone or combination for 48 hours and cells were stained with annexin V/PI dual staining and analyzed by flow cytometry. In MDA-MB-231 cells, olaparib or embelin alone induced 14.4 ± 1.1% or 17.1 ± 0.6% apoptosis, respectively, whereas co-treatment with olaparib and embelin exposure significantly increased the apoptotic population to 50.7 ± 1.5% (*p* < 0.05). A similar synergistic effect was also observed in CAL-120 cells (Figure 2D).

### Olaparib but not embelin activates caspase-8 mediated extrinsic apoptotic signaling pathway in BC cells

**Figure 3:**
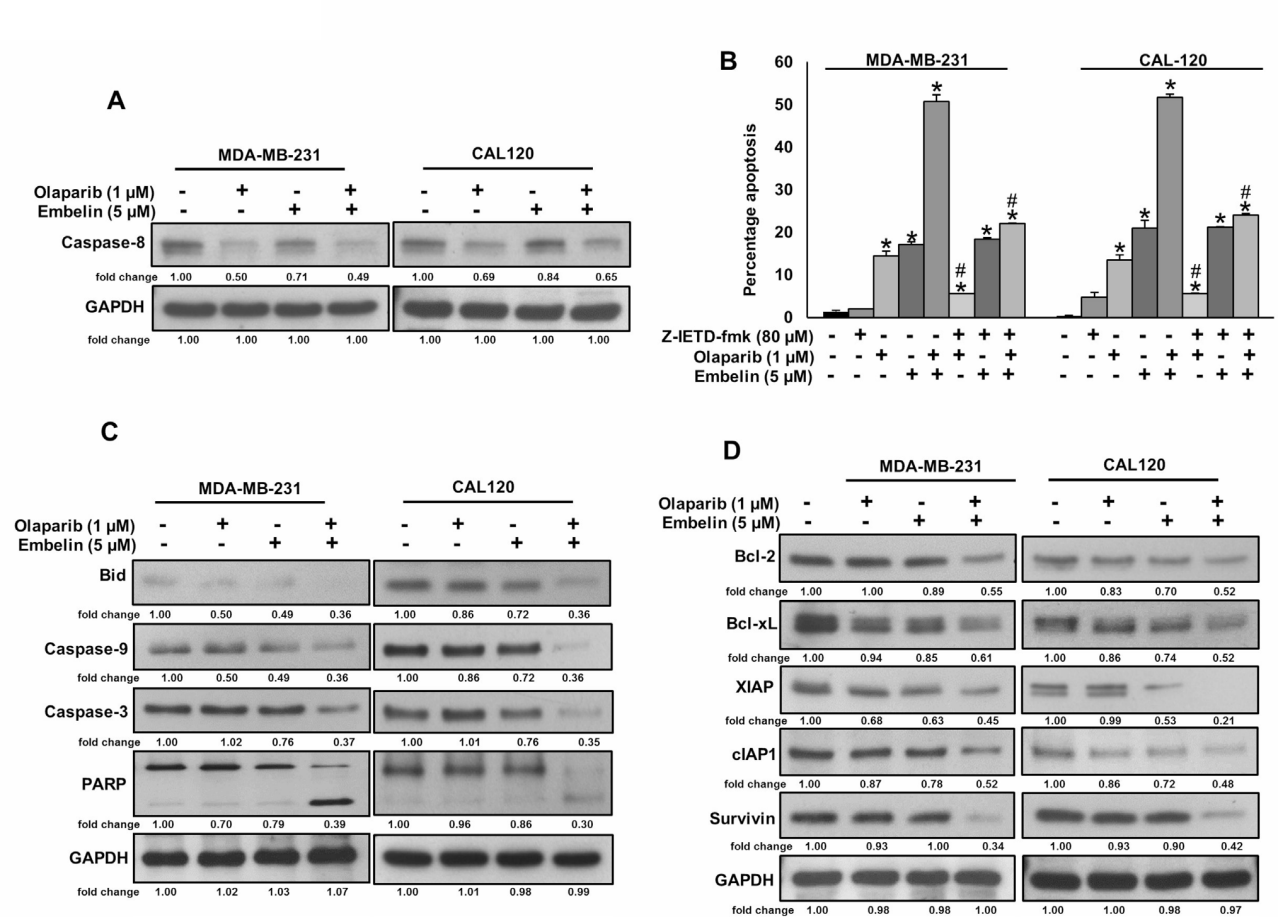
Olaparib but not embelin activates caspase-8 mediated extrinsic apoptotic signaling pathway in BC cells (**A**) Effect of olaparib and embelin on cleavage of caspase-8 in BC cells. BC cells were incubated with indicated doses of olaparib and embelin either alone or combination for 48 hours. After cell lysis, equal amounts of proteins were separated by SDS-PAGE, transferred to immobilon membrane, and immuno-blotted with antibodies against caspase-8 and GAPDH as indicated. (**B**) Effect of caspase-8 inhibition on olaparib and embelin induced apoptosis in BC cells. BC cells were pre-treated with caspase-8 inhibitor, z-IETD-fmk for 3 hours and subsequently treated with olaparib and embelin either alone or combination for 48 hours and cells were stained with flourescein-conjugated annexin-V and propidium iodide (PI) and analyzed by flow cytometry. Data presented in bar graphs are the mean ± SD of three independent experiments. ^*^and # indicate statistically significant differences compared to control without treatment or olaparib/embelin/combination treatment, respectively with *p* < 0.05. (**C**) Effect of olaparib and embelin on truncation of Bid and activation of caspase cascade in BC cells. BC cells were incubated with indicated doses of olaparib and embelin either alone or combination for 48 hours. Thereafter, the cells were lysed and proteins were immunoblotted with antibodies against Bid, Caspase-9, Caspase-3, PARP and GAPDH. (**D**) Effect of olaparib and embelin on the inhibition of anti-apoptotic proteins expression in BC cells. BC cells were incubated with indicated doses of olaparib and embelin either alone or combination. After 48 hours, cells were lysed and proteins were immunoblotted with antibodies against Bcl-2, Bcl-xl, XIAP, cIAP1, Survivin and GAPDH.

**Figure 4:**
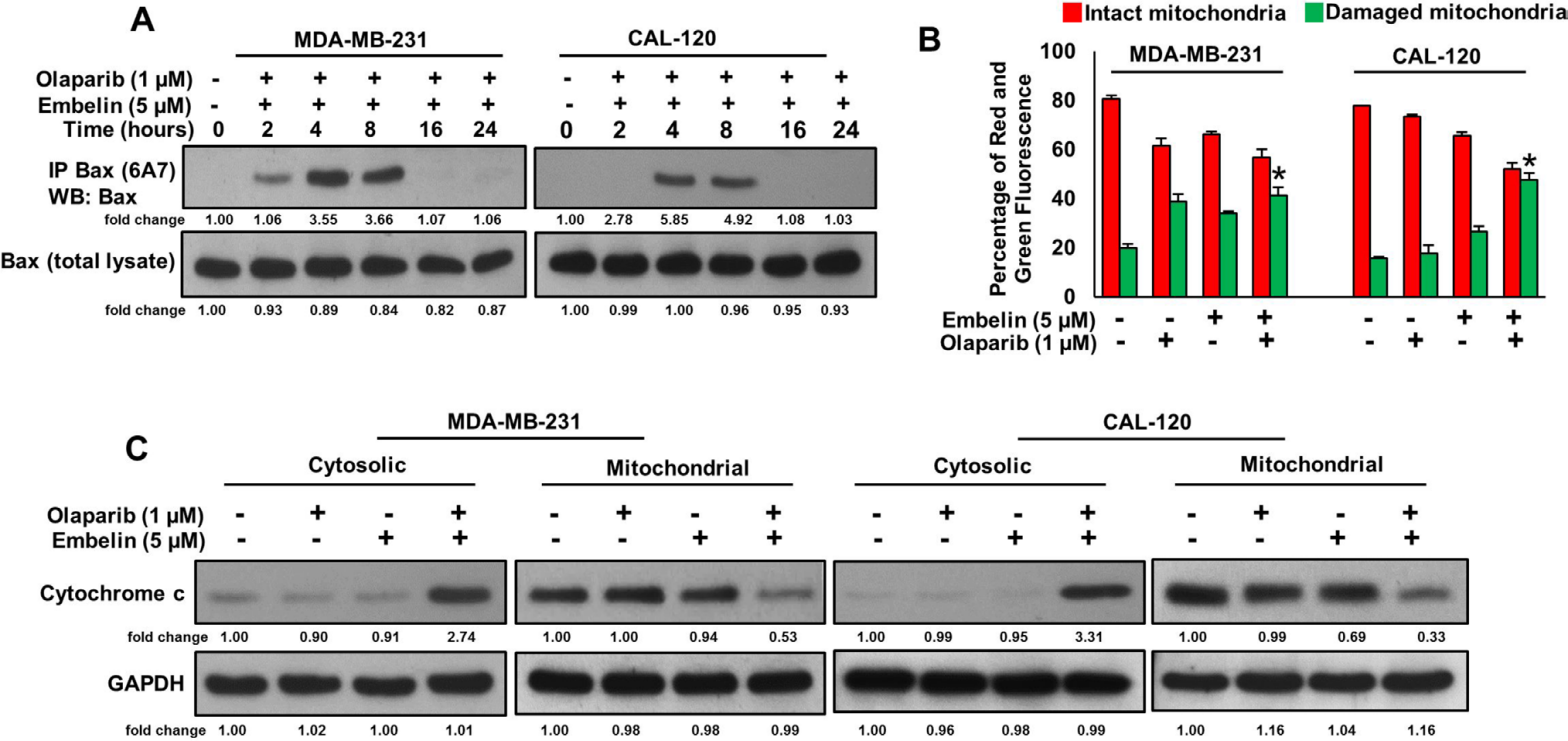
Olaparib and embelin synergistically induces mitochondrial apoptotic pathway in BC cells (**A**) Effect of olaparib and embelin on Bax activation in BC cells. BC cells were cotreated with olaparib and embelin for indicated time periods. Following treatment, cells were lysed in 1% Chaps lysis buffer and subjected to immuno-precipitation with anti-Bax 6A7 monoclonal antibody and probed with specific polyclonal anti-Bax antibody (top band) for detection of conformationally changed Bax protein. In addition, the total cell lysates (bottom band) were applied directly to SDS–PAGE, transferred to immobilon membrane and immuno-blotted with specific anti-Bax polyclonal antibody. (**B**) Effect of olaparib and embelin on mitochondrial membrane potential. BC cells were treated with and without indicated doses olaparib and embelin either alone or combination for 48 hours. Cells with intact mitochondrial membrane potential (red bar) and with lost mitochondrial membrane potential (green bar) was measured by JC-1 staining and analyzed by flow cytometry as described in Materials and Methods. Data presented in bar graphs are the mean ± SD of three independent experiments. ^*^indicate statistically significant differences compared to control without treatment with *p* < 0.05. (**C**) Effect of olaparib and embelin on cytochrome-c release. BC cells were treated with olaparib and embelin either alone or combination for 48 hours. Mitochondrial free cytoplasmic as well as mitochondrial fractions were isolated as described in Materials and Methods. Cell extracts were separated on SDS-PAGE, transferred to PVDF membrane, and immuno-blotted with an antibody against cytochrome c and GAPDH.

Since caspase-8 plays a pivotal role in the extrinsic apoptotic signaling pathway [32], we investigated whether treatment of olaparib and embelin causes activation of caspase-8 and Bid truncation in BC cells. BC cells were treated with olaparib (1 μM) and embelin (5 μM) either alone or combination for 48 hours and western blot analysis was performed using antibodies against caspase-8. As shown in Figure 3A, treatment of olaparib markedly activated caspase-8, whereas embelin has no effect on caspase-8 activation. In order to confirm the role of caspases-8 in olaparib and embelin induced apoptosis, BC cells were pre-treated with specific caspase-8 inhibitor, z-IETD-fmk (80 µM) for three hours followed by treatment with olaparib, embelin and combination for 48 hours. As shown in Figure 3B, there was appreciable inhibition of apoptosis in z-IETD-fmk pre-treated cells as compared to olaparib and embelin co-treated cells alone. In addition, caspase-8 inhibition by z-IETD-fmk significantly inhibited olaparib induced apoptosis, whereas z-IETD-fmk has no effect on embelin induced apoptosis (Figure 3B). These data clearly indicated that olaparib induces caspase-8 mediated apoptosis but not embelin in BC cells. Treatment with olaparib and embelin synergistically induced activation and cleavage of Bid, Caspase-9, Caspase-3 and PARP in both cell lines tested (Figure 3C). Truncated Bid translocate to the mitochondrial membrane to activate Bax or Bak and inactivate anti-apoptotic proteins such as Bcl-2 and Bcl-xl, resulting in the release of cytochrome c [33, 34]. Co-treatment of olaparib and embelin truncated Bid and down-regulated the expression of Bcl-2 and Bcl-Xl in both the cell lines tested (Figure 3D). Olaparib and embelin synergistically down-regulated the inhibitor of apoptosis proteins (IAPs), cIAP1, XIAP and Survivin, that play an important role in inhibition of apoptosis (Figure 3D). We then determined the effect of truncation of Bid on Bax activation. We co-treated with olaparib and embelin for different time periods in BC cell lines. We found evidence that Bax protein underwent conformational changes at 8 hours in both BC cell lines (Figure 4A). We next determined whether conformational changes in Bax protein caused change in mitochondrial membrane potential, the early event of activation of mitochondrial apoptotic pathway in BC cells. BC cells were treated with olaparib and embelin either alone or combination for 48 hours. Following treatment, cells were stained with JC1 dye for detection of loss of mitochondrial membrane potential by flow cytometry. As shown in Figure 4B, co-treatment of olaparib and embelin caused loss of mitochondrial membrane potential as measured by JC1 stained green florescence depicting apoptotic cells in both cell lines tested leading to release of cytochrome c from mitochondria into cytosol (Figure 4C). These results suggest that olaparib activates caspase-8 mediated extrinsic pathway whereas embelin induces intrinsic apoptotic signaling pathway in BC cells.

### Olaparib and embelin synergistically inhibits MDA-MB-231 xenograft growth *in vivo*

**Figure 5:**
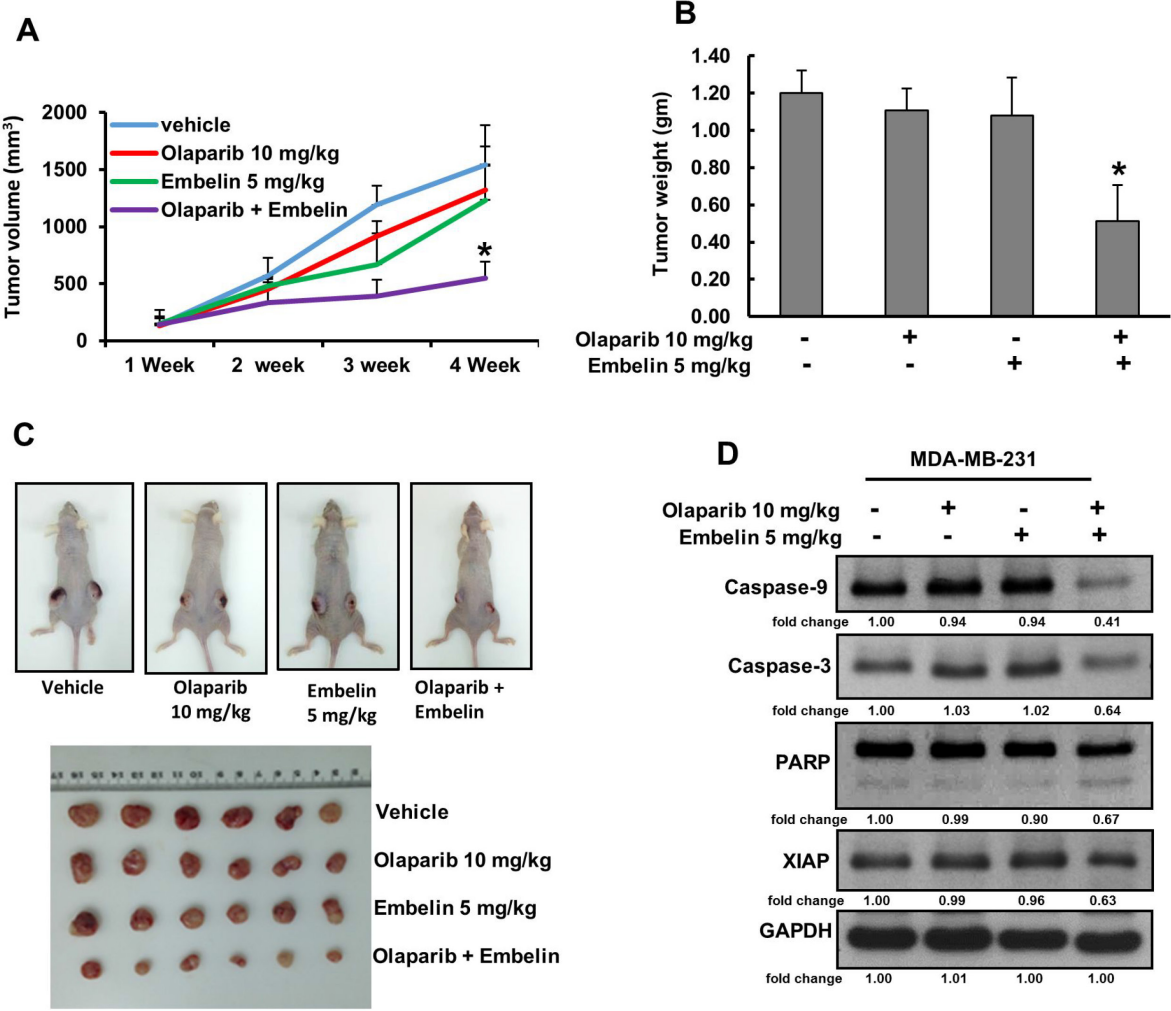
Olaparib and embelin synergistically inhibits the growth of MDA-MB-231 xenografts Female nude mice 6 weeks of age were injected subcutaneously with 4 × 10^6^ MDA-MB-231 cells. After one week, mice were treated intraperitoneal with olaparib (10 mg/kg), embelin (5 mg/kg) and combination twice a week for 30 days. DMSO (5%) in PBS served as a vehicle control. (**A**) Volume of each tumor was measured every week and the average (*n* = 5) tumor volume of mice was calculated, ^*^p < 0.05 inhibition of BC tumor growth by combination of olaparib and embelin treatment. (**B**) After 4 weeks of treatment, mice were sacrificed and tumor weights were measured, ^*^p < 0.05 compared to vehicle-treated mice by Student’s *t*-test. (**C**) Representative tumor images of vehicle, olaparib, embelin and combination of olaparib and embelin treated mice. (**D**) Whole cell lysate from mice treated with vehicle, olaparib, embelin and combination of olaparib and embelin were isolated and 10 µg protein were separated by SDS-PAGE, transferred to PVDF membrane, and immunoblotted with antibodies against Caspase-9, Caspase-3, PARP, XIAP and GAPDH.

Combination of olaparib and embelin induced synergistic apoptotic response *in vitro*. Therefore, we sought to determine whether this combination of treatment will result in anti-cancer effect *in vivo*. For xenograft study, MDA-MB-231 cells (4 × 10^6^ cells per mouse) were subcutaneously injected into the flanks of nude mice. After 1 week of inoculation, mice were randomly assigned into four groups: The first group received 0.9% normal saline as vehicle control while the other three groups received olaparib (10 mg/kg), embelin (5 mg/kg) and combination of both, injected twice weekly, intra-peritoneally. After 4 weeks’ treatment, mice were sacrificed and tumors were collected. There was no significant change in the tumor volume after treatment with olaparib and embelin alone but combination of both the drugs reduced the tumor volume within 3 weeks of treatment and reached significance at the end of the fourth week (Figure 5A). The weight of the tumor also reduced significantly following co-treatment of olaparib and embelin as compared to independent treatments (Figure 5B). Images of tumor showed that co-treatment of olaparib and embelin caused shrinkage of tumor size (Figure 5C). Finally, proteins isolated from tumors showed down-regulation of XIAP as well as cleavage of caspase-9, caspase-3 and PARP following co-treatment with olaparib and embelin indicating that co-targeting PARP and XIAP can regress BC xenografts in nude mice (Figure 5D).

## DISCUSSION

PARP mediates DNA single and double strand break repair through BER pathway [35–37]. Over-expression of PARP has been noted in several cancers, including breast cancer [9, 38–43]. To our knowledge, this is the first report on PARP protein expression in a large cohort of primary Middle Eastern BC by immunohistochemistry in a tissue microarray format. We show that high nuclear PARP (nPARP) expression was detectable in 44.7% of the examined cases. nPARP expression was, in general, correlated with more aggressive tumor characteristics. The aggressive TNBC cases showed the highest frequency of nPARP. Furthermore, high nPARP expression was significantly associated with poor overall survival even in multi-variate analysis. Our findings are similar to a study by Rojo *et al.*, who showed a poor overall survival in early breast cancer cases with over-expression of nPARP [9]. Donizy *et al.* also reported that over-expression of PARP was an unfavorable prognostic marker [44]. The patient poor survival in our cohort worsened significantly if the tumors co-expressed both PARP and XIAP, which could suggest that these two genetic deregulations might synergistically affect the survival outcome of breast cancer in this ethnicity.

Several cancers like TNBCs and ovarian cancers with mutant BRCA1 exhibit sensitivity to PARP inhibitors [45–49]. On the basis of our clinico-pathological observations, we hypothesized that co-targeting of both PARP and XIAP might be an affective therapeutic target in treating aggressive BC and might extend the utility of olaparib beyond *BRCA* mutant breast cancer patients. In the present work, we explored if PARP inhibitors might be extended beyond *BRCA*-deficient BC. We provide evidence that PARP inhibition leads to decreased cell viability through apoptosis in *BRCA* proficient BC cells. To gain more insight into the molecular mechanisms involved in decreasing viability following treatment, we investigated several representative apoptosis markers and found that PARP inhibition causes apoptosis through induction of caspase 8. The finding that caspase 8 inhibition significantly inhibited olaparib induced apoptosis further supports the notion that PARP inhibition could induce extrinsic apoptotic signaling pathway in BC cells.

Since we have previously demonstrated the effect of XIAP inhibition on BC cells [21] and the fact that our clinical samples showed that XIAP overexpression does significantly worsen the overall survival of patients who are overexpressing PARP protein, we sought to examine the effect of combining olaparib and embelin on BC cell viability and apoptosis. We observed that the cytotoxic effect of PARP and XIAP inhibition, administered in a sequential combination regimen was superior to PARP inhibition alone in downregulating survival and inducing apoptosis in *BRCA* proficient BC cell lines. Interestingly, the combination treatment of olaparib and embelin significantly reduced tumor growth *in vivo* when compared to treatment with each agent alone.

In summary, our result showed that PARP overexpression is a poor prognostic marker in Middle Eastern BC. *In vitro* data suggests that XIAP inhibition by natural compound, embelin, improved the response to PARP inhibition by olaparib and serves as a guide to translate this to substantial anti-tumor activity *in vivo*. The data improves our understanding of the roles that PARP and XIAP play, as well as support the clinical development of combined inhibition. This combined targeted approach might expand the role of PARP inhibition therapy beyond *BRCA*-deficient BC in the future.

## MATERIALS AND METHODS

### Patient samples and data collection

One thousand and eight patients with BC diagnosed between1990 and 2011 were selected from the files of the King Faisal Specialist Hospital and Research Centre (KFSHRC). The patients included in this study had their diagnosis, treatment and follow-up care in the Department of Surgical Oncology at KFSHRC. The histologic subtype of each breast tumor sample was determined according to World Health Organization (WHO) criteria. Detailed clinico-pathological data, including follow-up data, were noted from case records and summarized in Table 3. Waiver of consent was obtained for the study from the Institutional Review Board (IRB) and Research Ethics Committee (REC) of KFSHRC under Project RAC number 2170 021 on BC archival clinical samples. All samples were analyzed in a tissue microarray (TMA) format.

**Table 3:** Clinico-pathological variables for the patient cohort (*n* = 1008)

Age	*n* (%)
Young age (≤50)	686 (68.1)
Old age (>50)	322 (31.9)
Median (in years)	45.0
Range(IQR)^	39.0 – 54.0
**Histological type**	
Infiltrating Ductal carcinoma	917 (90.9)
Infiltrating Lobular carcinoma	46 (4.6)
Mucinous carcinoma	16 (1.6)
Others	29 (2.9)
**TNM Stage**	
I	87 (8.6)
II	407 (40.4)
III	381 (37.8)
IV	91 (9.0)
Unknown	42 (4.2)
**Histologic Grade**	
Well differentiated	81 (8.0)
Moderately differentiated	511 (50.7)
Poorly differentiated	405 (40.2)
Unknown	11 (1.1)
**Triple Negative Breast Cancer**	
Yes	149 (14.8)
No	851 (84.4)
Unknown	8 (0.8)
**Survival Duration (in months)**	
Median	48.0
Range(IQR)^	26.0–74.0

### Tissue microarray (TMA) construction

TMA construction was performed as described earlier [50]. Briefly, tissue cylinders with a diameter of 0.6 mm were punched from representative tumor regions of each donor tissue block and brought into recipient paraffin block using a modified semiautomatic robotic precision instrument (Beecher Instruments, Woodland, WI). Two cores of BC were arrayed from each case.

### Immunohistochemistry (IHC) staining and evaluation

Standard protocol was followed for IHC staining. For antigen retrieval, Dako (Dako Denmark A/S, Glostrup, Denmark) Target Retrieval Solution pH 9.0 (Catalog number S2368) was used, and the slides were placed in Pascal pressure cooker at 120°C for 10 minutes. The primary antibodies used for staining tissue microarray sections and their dilutions are listed in Supplementary Table 1. The Dako Envision Plus System kit was used as the secondary detection system with 3, 30-diaminobenzidine as chromogen. All slides were counterstained with haematoxylin, dehydrated, cleared and mounted. Negative controls included omission of the primary antibody. Normal tissues of different organ system were also included in the TMA to serve as control. Only fresh cut slides were stained simultaneously to minimize the influence of slide aging and maximise reproducibility of the experiment.

PARP scoring was done as described previously using the quickscore (QS) method [38]. Briefly, the proportion of positive cells was scored on a scale from 1 to 6 (1 = 1–4%; 2 = 5–19%; 3 = 20–39%; 4 = 40–59%; 5 = 60–79%; and 6 = 80–100%). The intensity of the positively staining cells was given a score from 0 to 3 (0 = no staining; 1 = weak, 2 = intermediate, and 3 = strong staining). QS was calculated by multiplying the percentage score by the intensity score, to yield a final score ranging from 0–18. Based on the QS, nuclear PARP-1 expression was graded as low (0–9) or high (10–18). Other IHC markers such as XIAP and Ki67 were scored as described previously [22, 51, 52].

### Cell culture

BC cell lines, CAL-120 and MDA-MB-231 were purchased from American Type Culture Collection (ATCC) and grown in RPMI 1640 media supplemented with 10% fetal bovine serum (FBS), 100 Units/ml penicillin/streptomycin and 100 Units/ml glutamine. Cells were cultured at 37°C under a humidified 95%: 5% (v/v) mixture of air and CO_2_. Both cell lines were authenticated in house using short tandem repeats PCR and the results (Supplementary Table 2) were in concordance with published data [53, 54]. All experiments were performed using 5% FBS in RPMI 1640 media.

### Reagents and antibodies

Olaparib was purchased from Selleck Chemicals (Houston, TX). Embelin was purchased from Tocris Bioscience (Ellisville, MO). Antibodies against PARP, caspase-8, caspase-9, caspase-3, Bcl-2, Bcl-xl and cIAP1 were purchased from Cell Signaling Technologies (Beverly, MA). XIAP antibody was purchased from BD Pharmingen (San Diego, CA, USA). Survivin, Cytochrome c and GAPDH antibodies were purchased from Santa Cruz Biotechnology, Inc. (Santa Cruz, CA). Annexin V was purchased from Thermo Fischer Scientific (Waltham, MA). Caspase-8 inhibitor, z-IETD-FMK was purchased from R&D Systems (Minneapolis, MN, USA).

### Cell viability assay

10^4^ cells were incubated in triplicate in a 96-well plate in the presence or absence of indicated test doses of olaparib and embelin either alone or combination in a final volume of 0.20 ml for 48 hours. Cell viability was determined by MTT assay, as described earlier [55]. Replicates of 6 wells for each dosage including vehicle control were analyzed for each experiment.

### Annexin V staining

BC cells were treated with olaparib and embelin either alone or combination for 48 hours and then were harvested. The percentage apoptosis was measured by flow cytometry after staining with fluorescein-conjugated annexin-V and propidium iodide (PI) as described earlier [21].

### Cell lysis and immunoblotting

Following treatment, BC cells were lysed in phosphorylation lysis buffer containing 50 mM Hepes (pH 7.3), 150 mM NaCl, 1.5 mM MgCl_2_, 1.0 mM EDTA (pH 8.0), 100 mM NaF, 10 mM Na_2_H_2_P_2_O_7_, 200 µM Na_3_VO_4_ and 1X proteasome inhibitors (Roche pharmaceuticals, Basel, Switzerland). Following lysis, cells were spun at 14,000 rpm for 15 minutes at 4°C and protein amounts were measured using Bradford assay (Life Technologies). Equal amounts of protein were separated on SDS-Page and immunoblotted with different antibodies as described previously [56].

### Detection of Bax conformational changes

Detection of Bax conformation was performed as previously described [57]. In brief, cells were cotreated with olaparib and embelin for different time periods after which cells were harvested and washed with PBS and lysed with Chaps lysis buffer (10 mM HEPES (pH 7.4), 150 mM NaCl, 1% Chaps) containing protease inhibitors. Concentration of proteins was assessed by Bradford assay and 200 µg of total protein was incubated with 6 µg of anti-Bax 6A7 monoclonal antibody for 2 hours at 4°C. Following incubation, 25 µl of protein G-beads were added into the reaction and incubated at 4°C overnight on a shaker with gentle agitation. Following 4 washes in Chaps lysis buffer, samples were separated by SDS-PAGE, transferred and immunoblotted using N20 Bax polyclonal antibody (Santa Cruz, CA).

### Measurement of mitochondrial membrane potential and cytochrome c release

Following treatment with olaparib and embelin for 48 hours, BC cells were harvested and stained with JC1 dye (Thermo Fischer Scientific, Waltham, MA) for 30 minutes at 37°C and analyzed by flow cytometry. In the same experiment, cells were also fractionized into mitochondrial free cytosolic and cytosolic free mitochondrial fractions and separated on SDS-Page for immunoblotting as described previously [58, 59].

### Animals and xenografts study

Six-week-old female nude mice were obtained from Jackson Laboratories (Bar Harbor, ME) and maintained in a pathogen-free animal facility at least 1 week before use. All animal studies were performed in accordance with institutional guidelines. For xenograft study, MDA-MB-231 cells (4 × 10^6^ cells per mouse) were re-suspended in serum-free medium with matrigel basement membrane matrix (BD Biosciences) at a 1:1 ratio (total volume=100 μl) and subcutaneously injected into the flanks of nude mice. After tumors grew to about 100 mm^3^, mice were treated intraperitoneally with olaparib (10 mg/kg), embelin (5 mg/kg) and combination twice a week for 30 days. The body weight and tumor volume of each mouse was monitored weekly [55]. After 4 weeks’ treatment, mice were sacrificed and individual tumors were weighed, then snap-frozen in liquid nitrogen for storage.

### Statistical analysis

Contingency table analysis and chi-square tests were used to study the relationship between clinico-pathological variables and PARP expression. Overall Survival curves were generated using the Kaplan–Meier method, with significance evaluated using the Mantel-Cox log-rank test. The limit of significance for all analyses was defined as *p* value of < 0.05; two-sided tests were used in these calculations. The JMP10.0 (SAS Institute, Inc., Cary, NC) software package was used for data analyses.

For all functional studies, data presented are means ± SD of triplicates in an independent experiment, which was repeated for at least two times with the same results. For multiple comparisons, one-way analysis of variance (ANOVA) was performed using IBM SPSS Statistics 21 software (IBM Corp., Armonk, NY). Values of *p* < 0.05 were considered statistically significant.

## SUPPLEMENTARY MATERIALS FIGURES AND TABLES


