# Supplementary material for: Overexpression of PARP is an independent prognostic marker for poor survival in Middle Eastern breast cancer and its inhibition can be enhanced with embelin co-treatment
Source: Oncotarget. 2018 Dec 18;9(99):37319–32. doi: 10.18632/oncotarget.26470 (PMC6324669; doi:10.18632/oncotarget.26470)
Supplement: Supplementary file 1 [file oncotarget-09-37319-s001.pdf]

Overexpression of PARP is an independent prognostic marker for poor survival in Middle Eastern breast cancer and its inhibition can be enhanced with embelin co-treatment

SUPPLEMENTARY MATERIALS

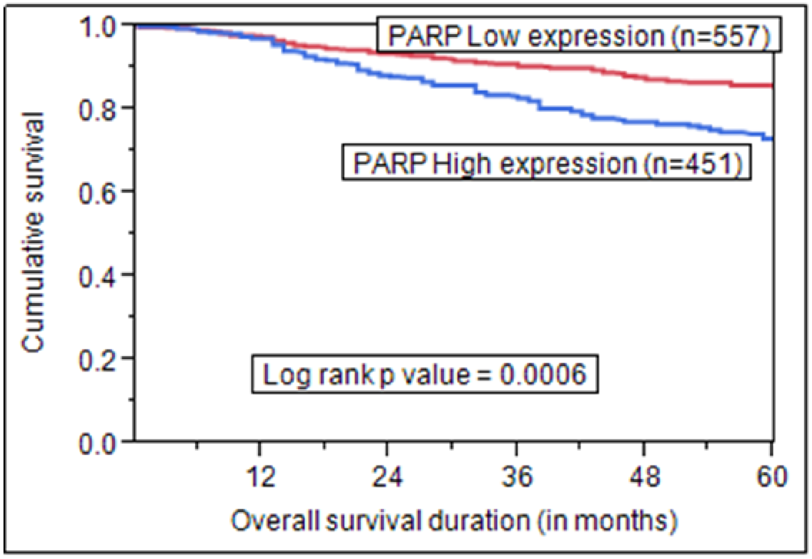

Supplementary Figure 1: Kaplan–Meier survival analysis for the prognostic significance of PARP expression in breast cancer. Breast cancer patients with overexpression of PARP had reduced overall survival at 5 years compared to tumors showing low expression of PARP (p = 0.0006).

MDA-MB-231

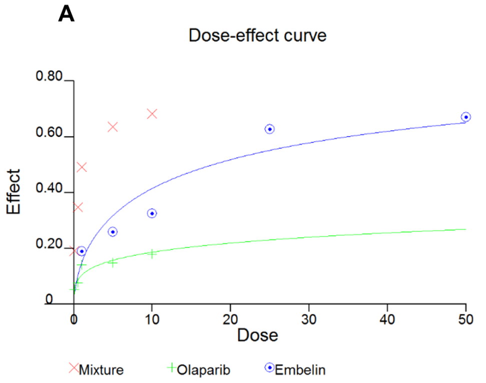

MDA-MB-231

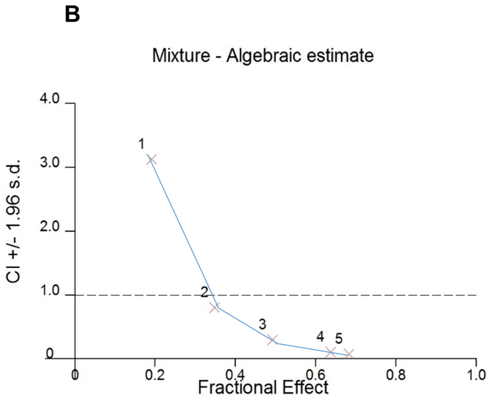

C

Combination index (CI) for experimental values			
**Olaparib (μM)**	**Embelin (μM)**	**Fa**	**CI**
0.1	5	0.191	3.126
0.5	5	0.348	0.809
**1**	**5**	**0.492**	**0.298**
5	5	0.637	0.111
10	5	0.682	0.079

Supplementary Figure 2: Synergistic inhibition of cell viability by olaparib and embelin in MDA-MB-231 cells. MDA-MB-231 cells were treated with various combinations of olaparib and embelin for 48 h and dose effect (**A**) and Fractional effect (**B**) graphs were generated using Calcusyn software. (**C**) Combination Index (CI) were calculated using Chou and Talalay method.

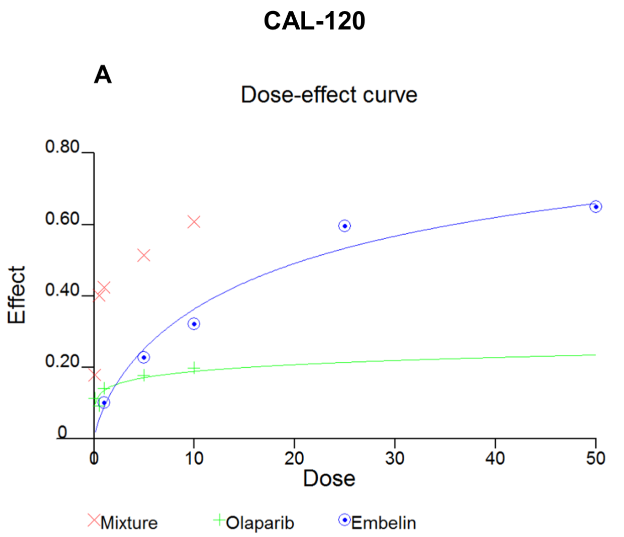

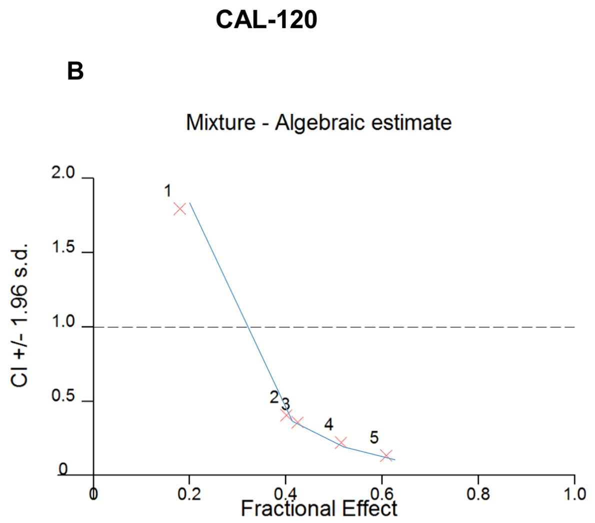

C

CAL-120

**Combination index (CI) for experimental values**			
**Olaparib (μM)**	**Embelin (μM)**	**Fa**	**CI**
0.1	5	0.1796	1.798
0.5	5	0.4017	0.405
**1**	**5**	**0.4243**	**0.358**
5	5	0.5149	0.221
10	5	0.6083	0.134

Supplementary Figure 3: Synergistic inhibition of cell viability by olaparib and embelin in CAL-120 cells. CAL-120 cells were treated with various combinations of olaparib and embelin for 48 h and dose effect (**A**) and Fractional effect (**B**) graphs were generated using Calcusyn software. (**C**) Combination Index (CI) were calculated using Chou and Talalay method.

Supplementary Table 1: Antibodies used for TMA IHC analysis

Antibody	Clone	Company	Source	Dilution*	Retrieval	Detection Kit
PARP	F-2	Santa Cruz Biotechnology	Mouse	1:300	pH 6	Envison +
XIAP	48	BD Biosciences	Mouse	1:400	pH 9	Envison +
Ki-67	Mib-1	Dako	Mouse	1:500	pH 9	Envison +

*Overnight incubation.

Supplementary Table 2: STR profile for MDA-MB-231 and CAL-120 cells

**MDA-MB-231**	
Markers:	
Amelogenin	X
CSF1PO	12, 13
D13S317	13
D16S539	12
D18S51	11, 16
D19S433	11, 14
D21S11	30, 33.2
D2S1338	20, 21
D3S1358	16
D5S818	12
D7S820	8, 9
D8S1179	13
FGA	22, 23
TH01	7, 9.3
TPOX	8, 9
vWA	15, 18
**CAL-120**	
Markers:	
Amelogenin	X
CSF1PO	12
D13S317	12
D16S539	12
D18S51	12
D21S11	30
D3S1358	18
D5S818	13
D7S820	12
D8S1179	12, 13
FGA	21
TH01	6
TPOX	8, 9
vWA	18
